# Educating the Dentist*Read before the Odontological Society of Chicago, April 9, 1912

**Published:** 1912-09-15

**Authors:** A. E. Royce

**Affiliations:** Chicago, Ills.


### EDUCATING THE DENTIST.*

BY A. E. ROYCE, D.D.S., CHICAGO, ILLS.

The most important subject before the dental profession today is the dentist, for upon him depends the whole future of the profession. As he rises in the estimation of the people, so the profession as a whole is placed upon a higher plane. As the standard of his education is advanced and his mind broadened so that he becomes more of a power, just so much the profession gains in the opinion of the community in which he as an individual resides. With this in mind I repeat that the most important subject before the dental profession today is the dentist. And what shall be the course of the special training he will have to undergo in order to best fit him to perform his part in the advancement of himself and the profession? Shall he be obliged to go to medical schools for the instruction required, or will a preliminary education of high school and university work, together with the course in our own dental colleges best equip him to gain skill and prominence in his life work? Now, I do not propose to give you a dissertation on dental education, but there has been so much agitation of late in regard to the necessity of a medical degree for the dentist, and even a question of enacting a law (as Virginia has) by which such a degree is made obligatory, that I feel impelled to present some of the undesirable features and some of the burdens such a law would entail upon the average dental student—those not possessed of a large amount of this world's goods, but who seek to equip themselves in the best possible manner without too great an outlay of time and money.

There are two distinct sides to dentistry, the theoretical and the practical, and neither of them can be neglected. I need not go into history and tell you of the triumphs of

*Read before the Odontological Society of Chicago, April 9, 1912

the American dentist abroad, because of his dexterous fingers, but while finger skill is most important, he must now have more than that for the scope of dentistry has extended until a dentist must have a training in anatomy, chemistry, bacteriology, physiology, pathology, metallurgy, therapeutics, etc., etc., sufficiently broad so that he can with intelligence meet all the demands made upon his skill in the present day practice of his profession. Not only that, but he should also be so fitted for life that he may take a proper social position so that his influence in the community and as a citizen may be fitting to his high calling. I say "high calling," for I believe that proper care of the mouth and teeth is as important to the health and happiness of the human race as any other one thing.

The changes in the course of dental instruction that have taken place within the memory of those present have been very marked, and with increased knowledge of dental subjects and needs, there is a growing demand for a further step forward particularly where dentistry reaches over into the domain of medicine, and it is but natural the cry should go up that the medical degree is essential to the successful practice of dentistry. It is my observation (generally speaking) that the men who advocate the M. D. belittle the operative side of dentistry; it is not unusual to hear them say that any jeweler or watchmaker can do these operations, that it is purely mechanical and requires no particular knowledge except the ability to bore holes and fill them. Now, this class of labor, to do the mechanical work, can be employed very cheaply and with medical men at the head of the office to receive the patients and decide upon the operations to be performed, and incidentally pocket the money, it is easy to see how they could subordinate the dental profession until it would in time become one of the lost arts. This picture may seem overdrawn, but it ts only a legitimate conclusion drawn from statements made by men advocating the necessity of the medical degree for the practice of dentistry.

The dental colleges seem always to be a mark for criticism, some of which no doubt is justified for the men at the head of our schools are only human and must learn by their mistakes, just as the rest of us do, but much that is said bears the marks of prejudice, if not malice. Our schools have come up from a small beginning, and in the face of much opposition from the physicians until they now stand accredited institutions in our country, and they deserve a great deal of credit for what they have done. They have always stood ready to advance the standard of education as fast as the requirements arose, and I believe it will not be many years before other cities than Boston will have their dental laboratories for technical and scientific research. At the present time our schools are preparing men for the general practice of dentistry just as the medical schools are fitting their students for the general practice of medicine; if either a physician or dentist desires to follow a specialty he must take special courses to fit himself for that work. It is not necessary for the general practitioner of medicine to be trained in dentistry, but it is necessary for him to be able and willing to recognize a condition in the mouth which should be referred to a dentist and upon which the future welfare of the patient may hinge; neither is it necessary for a dentist to be trained in medicine, but he should be able to treat the diseases that come within his province and also to recognize cases that should be sent to a physician. The physicians are coming more and more to appreciate the necessity of caring for the mouth and teeth because of their influence on the general health, but I believe today there is more harm done through ignorance of dentistry on the part of the general practitioner of medicine than because the dentist has not taken the medical degree.

A few years ago all the dental colleges connected with the Dental Faculties Association increased their course to four years in the endeavor to make the course more comprehensive, but the number of the students was very materially decreased and the fact that the colleges have now returned to the three years' course has been attributed to this reason.

The present campaign of educating the people to the necessity of dental work, and the proposed dental work in the schools, will increase the demand for the services of dentists and to meet this we must increase our student body rather than decrease it by making the course too long. It is freely stated that the dentists of Michigan, Indiana and Illinois could not, at the present time, properly take care of the teeth of the school children of Chicago. Our colleges will tell you that there is a constant demand being made upon them for dentists. Small towns write and ask that a dentist be sent to them, as the people are in need of his services, and there is none within ten, fifteen or twenty-five miles. This shows a scarcity of dentists throughout the country that is appalling.

Suppose we consider for a moment what the effect would be if the entrance requirements for our colleges were a full medical course. The best medical schools of the present day require two years' scientific work in a university, then the medical course of four years and the dental college would require two years more, making eight years of work after the young man left high school before he would be considered capable of conducting a dental practice. There are some very strong arguments against this, one of them being the impossibility of a student of, say, twenty-five years of age, to acquire the manual dexterity necessary to maintain the high operative standard of the past. It must be remembered that it is the finger skill of the American dentist that has given him his place in the world, and that skill should be maintained and guarded with the utmost care. Again, the shorter average life of the dentist proves it is more trying than any other profession. It is also less remunerative than most of the specialties of medicine. How many young men—graduate physicians—would pass by the advantages these specialties offer and take up the study of dentistry. Many of the young men who enter our dental schools have but limited means with which to acquire their education. This class of men are usually earnest, hard-working fellows who later in life are a credit to the profession. Is it not laying upon them a greater burden than is necessary to make the course eight years after high school? If the requirements for entrance to the dental schools is made equal to that of the best medical schools and that portion of medicine needed by the dentist in his practice is taught as thoroughly as it is in the medical schools, the dental graduate should be as well equipped for the practice of his profession as if he had taken a complete medical course; in fact, I have often thought the acquiring of medical knowledge induces a neglect of details that are essential to secure the best results in dental operations. One of the great difficulties found in all professional schools is that the students do not know how to study. I am told that since the medical schools have required two years in university their students study much more intelligently—they better appreciate the necessity and value of the subjects they are trying to master, and for that reason do far better work than when they went from high school into medical college. The same would prove true in our dental schools, our young men lack in concentration and application—they don't know how to study—but give them two years in a university and they will have not only the broadening effect of the university work, but they will bring an intelligence, earnestness and concentration to their work which will enable them to grasp the best that the college can offer them.

Thinking to find out how other dentists viewed this subject, I have had some correspondence with a number of the best men in the state, men who stand high in the dental societies and whose ideas are foremost for the advancement of the profession; some of them also hold the degree of M. D. as well as D. D. S., and I am pleased to say their thought along this line is much the same as my own—all standing for higher education, and all thinking an eight-year course is longer than we should oblige our students to take. They all agree that the educational standard of our dental students should be raised, but that it should be a preliminary and university course rather than an obligatory medical course. It would be ideal to have an education in medicine and it would be ideal to be an expert surgeon and chemist and bacteriologist, but it is not practical —practically we must educate our students to be general practitioners and it is time the rank and file of the dentists roused themselves to the fact that the dental profession is likely to pass out of their control.

### DISCUSSION.

Dr. C. S. Case: I have never considered that a medical degree should be regarded as absolutely important or necessary for a dentist to possess, but like other great branches of education it would help to broaden a man's ideas and give him a better education and a better understanding, because in taking that course he is obliged to go through certain studies and acquaint himself in certain branches that he would not do if he was left to himself. In that way it is certainly a great advantage to him.

I attribute largely the success I have had in my special branch of dentistry to the fact that I took the medical degree, and because I was obliged to fully acquaint myself with those branches which lie at its foundation. If dentists would take up other branches of study such as biology and geology and chemistry they would certainly improve and broaden themselves.

Dr. C. N. Johnson: I want to express my very high appreciation of Dr. Royce's paper and to say that I am in thorough accord with the sentiments expressed therein. I believe that Dr. Case has probably misunderstood the purport of Dr. Royce's paper in one respect. Dr. Case took his medical degree after he took his dental degree.

I venture to make the assertion tonight that if Dr. Case had taken his medical degree and then his dental degree he would not be the great dentist that he is today. I say this advisedly in view of my wide experience in dealing with men in dental colleges who have been graduates of medicine in advance of their dental degree. They come to us, as Dr. Royce has intimated in his paper, with an exalted idea of the value of medicine and a very limited outlook so far as the value of the dental course is concerned. They have the wrong point of view, so far as the making of practical dentists is concerned, and I say a man who has been a medical graduate and then graduated in dentistry and made a successful dentist and is brilliant in his profession as a dentist, is so because of his inherent ability and a natural tendency toward mechanical pursuits. Our experience, and we have had a great many medical men in the college in which I teach, has been that they come to us with the idea that dentistry is a mere side issue and simply an appendix of medicine, with very little to learn after the medical course. I do not wish to seem to be in favor in any way of restricted education. I believe in producing the best possible dentist in our schools. I believe if we could get manual training in advance of our professional training it would be beneficial. A man's manipulative skill is one of the essential factors, and if you put him in the common school, and then in the high school and then to the university, it is barely possible that boy has lost the adaptability of his fingers to make a good manipulative dentist. We discuss this from a professional point of view forgetting that the important factor is the service to the people. You and I are here to serve. I am taking up and examining this question as I have never done before. I read a book recently by William Hawley Smith and this book goes into the fundamentals of this question. The whole system of education, dental, medical and common school, needs to be revised. In the early days these methods were the best methods we could use at that time; methods by which we could accomplish the best results as we saw it, but the profession has grown very rapidly and not only that, but the demand of the people has grown much more rapidly. I am the last man in the dental profession to wish to narrow in any way the preliminary requirements of our students, and I glory in the man who has graduated in dentistry and then has felt he wanted to broaden his knowledge and graduate in medicine. I honor that man as I would honor the man who studied biology to broaden himself, or chemistry, but usually the man who does the greatest service to the people is the one who has concentrated himself on some one thing just as Dr. Case has done in orthodontia. Dentistry is so broad that no one man can encompass it.

Under the Virginia law a man must be a medical graduate before he is a dentist. I predict that this will not result in the best possible dental service to the people, because many of the young men who take the medical and then the dental course will, before the dental course is reached, have lost their manipulative adaptability to the extent that they will never make good operators.

Dr. P. J. Kester: I have not been in college work lately, nor have I been in it as much as Dr. Johnson, but I can corroborate what he has already said about the medical man who takes up dentistry. I think we all know the difficulties that we have in training medical men in dental lines. I did not graduate in medicine, although I had two years in medicine and could have graduated in medicine when I graduated in dentistry, but I liked to graduate with the dentists because I had a dental training for several years, but I wished to get the general facts of the medical training with the man who takes medicine, entirely at first hand.

As to the time that a man should devote to his studies in dentistry, and as to the preliminary education, it seems to me that is a debatable question, just as I have raised the question with our past secretary of the Board of Dental

Examiners as to the examination of a man who comes before the board to secure a license to practice dentistry. State laws say a man to practice dentistry shall be a graduate of a dental college. Now I do not believe that is any business of the state board of examiners where a man gets his knowledge; if he can demonstrate he has the knowledge necessary to practice dentistry. So regarding the preliminary education I do not know. One of the very greatest men in scientific dentistry that I have personally known, was a man who never had but four months in a primary school. Some of the greatest operators and some of the most scientific men I have known are productions of the common schools in this country. I do not believe that a college education is necessary for the development of the dentist, except that in college he learns certain things that will help him, but they are not absolutely necessary. A man with a high or grammar school education can study dentistry as intelligently as the man who comes from Yale or Harvard. There is nothing in the study of dentistry that any ordinary intelligent man that is fairly developed from a common school is not able to comprehend. I think if a man wants to learn these things he can just as well as the man learned them before him; so, I do not believe that it is absolutely necessary for a man to have a college education to study dentistry. I believe, as has been suggested here, that it takes time to develop the dentist. Had I studied medicine, the probabilities are I might have looked upon dentistry as a sort of mechanical job, as nearly all of our medical friends do; there are very few of our medical friends, as far as I know, take them as a whole, that have very much regard for dentistry. They look upon it the same or very little different from the jeweler or blacksmith and that of other mechanics.

I believe that Dr. Johnson is right and that we need to revise our educational methods; we are in a transition state; we are leaving our old ideas of mind development; and the question is now whether they have got a system which will develop the youthful mind as well as the old fashioned country school that we all attended, and dentistry is the same to my mind to a certain extent.

Dr. Elliott R. Carpenter: Personally, I do not quite agree with the preceding speakers in the fact that the medical profession does not appreciate what the dental profession is doing. I think the attitude of Dr. Evans and your own personal family physicians would bear this out and discredit that statement. The medical profession has been forced to recognize that we are not merely higher mechanics and that we are delving very close along the lines they are and incidentally doing well.

The principal thing that can be said from my point of view for college education before the dental course is the fact of the mental training and of its teaching men how to study and make deductions. If we must have a compromise on that, let that college course be one of higher mechanics, mechanical engineering and that sort of thing, and we will then get the broadening effect, the mental training, and carry along the training of the fingers, and I hope something of that kind will ultimately come about.

Dr. George W. Cook: The essayist has touched upon the very essence of higher education for dentists; instead of a medical education, let it be a broader education of science and mechanics.

Many of the high school graduates that the dental colleges get are the poorest students. They may have studied botany, zoology, physiology, but when they get into college and see how much there is to do they get to work. and get a good scientific education in a dental college. Of course, qualification of a man is a great thing, but you must have the man in the proper shape to be qualified.

I think of all the men I have met, the dentist is the best trained thinker. He is able to concentrate his mind on a particular thing till his ingenuity becomes great. Do you suppose that Dr. Taggart would have been the inventor that he is today, would have accomplished as much if he were a medical college graduate? He would probably have known a little more anatomy or some particular subjects of science, but he would not have been the inventive genius that he is, he would not have developed that ingenuity. This is what has made dentistry—that concentration of thought, use of fingers and eyes that have made men see and know how to do things. The dentist learns to see and to do, and those are the most important things in educatiom to my mind. They are the very essence of education.

On this question of education requirements and examinations, I think we are trying to do too much. We are trying to do something it is impossible to do with the conditions as they are. Except in one or two instances, not a single contribution of money has anybody ever given towards dental education. Most men who have ever taught in dental colleges have done so at a great sacrifice. Men have built dental colleges in this country through their own ingenuity or manipulation of money affairs, and have given to the people the best it was possible at a great sacrifice. If the people want the standards raised let them do something towards it. Those who have been connected with the dental colleges are just as anxious to turn out good dentists as the people are to have good dentists. Of course I know that lots of men graduate that are not as well qualified as we could wish. I had two telegrams from a man in Michigan asking for a dentist; he lived in a town of 2,500 and had to ride thirty miles for a dentist and the dentists in the neighboring towns are so swamped it takes weeks to get an appointment. He said we need dentists and need them badly. When there is such a cry as that, are we going to deprive the people of such services, even though they are not the best?

Dr. J. E. Hinkins: I have long since been convinced of the fact that the educated man of today is not necessarily a college-bred man. There is quite a distinction between an educated man and a college-bred man in more ways than one. A few years ago when the school of Dr. Johnson and myself was organized, it required a medical education as a requisite before dental education, but after two or three years it was found to be a failure, because they were not turning out the class of men that the public wanted. They could not get remuneration for their services to pay for the investment necessary to acquire this education.

I do not think there was ever a time in the history of the world when there was a greater demand for the higher type of man than today in all walks of life. I have been very much interested in a series of articles in the transactions of the American Association for the advancement of science, by Dr. W. J. Spillman, who visited the charitable institutions in the United States, and submitted a report of what he saw was necessary for the betterment of the education of the masses and the betterment of the people at large. Mr. Spillman has shown that seventy percent of the efficient business men of the United States today are country-bred children; he has also shown that the country boys and girls are more efficient at the age of seven to nine, than the city boy or girl is at from thirteen to fifteen. He gives three reasons for that: First, that the child in the country is taught to do something; he is given some ground, or a pig, and that boy has got to produce something. The next was the advantage of fresh air, and, third, was the advantage of God's sunshine and mother earth. The country boy has a power which the city child can not acquire. There is something radically wrong somewhere with the method of education today. If I understand education it should make man of a higher type, morally and intellectually, but it does not seem to do so. Only last week I was at a meeting where the head man of the Y. M. C. A. was present and he had applications for young men to work. It represented a number of the leading business firms of Chicago and they wanted young men that were of good morals and good habits, and the letter closed by saying, "Send us boys with common school education and do not send us college graduates." That came from a number of firms in this city. That is the situation today of higher education. I think Mr. Crane well said that it was preposterous for any university or board of education, or school of education to say it could take a common mind and by higher education produce an uncommon mind. He said you could take a common mind and by higher education produce a very unsafe mind, but could not take a common mind and by higher education produce an uncommon mind. I believe today too many of our students are educated beyond their capacity.

Dr. J. G. Reid: As I see from this discussion we have not settled the question, and of course, it will never be settled, because our whole system of education is constantly changing. Within our own short lives we have passed through periods of education of various kinds and what is good today is not going to be good in ten years from now. What is good ten years from now is not going to be good twenty years from now.

You may cite a Lincoln, or you cite some eminent scientific man, or the greatest scientific men of the dental profession who may have had only four months of school education. What of it? One swallow does not make a summer. We did not educate the dental profession; we did not educate any one. One man did not do it, the citation of two men, or the citation of a hundred men did not do it. It is the citations of thousands of men that make progress in education. We had a Lincoln; well, now, we have not had any such man as that before nor since, but why? There was a peculiar environment, and a demand for something to be done at that particular time and he was the man that should do it. Do you not believe there are other men that are just as brilliant and if in the same position would have done the same thing and perhaps done it better, but the opportunity was not there?

Dr. Johnson criticises our whole system of education, but he did not suggest anything better. If we are constantly changing and going on we can't settle this question. We may have methods that are not perfect, but they have been a power for the good and for the betterment of education. There is such a diversity of educational opinions in dentistry and there are some dental colleges that are just as far behind in dental education today as they were twenty-five or thirty years ago when I went to college. There must be some controlling head somewhere. The dentist is a peculiar individual as a whole. I think I know a good many dentists that would be better off if they were not in the profession, and the dental profession would be better off without them —that is a great majority of them. It is a fact that there are too many poor dentists.

Dr. Royce (closing the discussion): I was led to write this paper because laws have been passed in several European countries and in the state of Virginia, that work injustice to the average dental student, for while we all believe a man cannot be too well prepared for the practice of his profession, should not that preparation be along the lines of future endeavor? A course can be formulated which will properly prepare a man for the practice of dentistry without obliging him to spend years in the study of medicine. The preliminary university course, which the medical schools require now, is a course which is really preliminary to the medical studies and assists medical students in their work very much. It is quite possible the preliminary course for the dentist will be somewhat different, as has been suggested tonight.

Some of you may remember a paper written by Dr. W. E. Royce of Tunbridge Wells, some years ago, in which he cites the necessity of beginning the training in early youth of any one who would become expert on a violin or piano, because it is absolutely impossible for one of mature years to gain the finger skill necessary to become expert on these instruments, and the rule holds good in dentistry.

The thing that impresses me in regard to students in dental colleges, is their tendency to make light of the scientific part of the work and their great ambition to put in fillings. With proper emphasis on the scientific work this could be avoided and the course so balanced as to turn out graduates well fitted for their work.—*Dental Review.*

